# Clinical entity-aware domain adaptation in low resource setting for inflammatory bowel disease

**DOI:** 10.3389/frai.2024.1450477

**Published:** 2025-01-14

**Authors:** Sumam Francis, Fernando Crema Garcia, Kanimozhi Uma, Willem Mestdagh, Bart De Moor, Marie-Francine Moens

**Affiliations:** ^1^Language Intelligence and Information Retrieval (LIIR) Lab, Department of Computer Science, KU Leuven, Leuven, Belgium; ^2^Dynamical Systems, Signal Processing and Data Analytics (ESAT-STADIUS), KU Leuven, Leuven, Belgium

**Keywords:** entity-aware pre-training, named entity recognition, clinical NER tool, contrastive learning, inflammatory bowel disease, language modeling, natural language processing, information extraction

## Abstract

The digitization of healthcare records has revolutionized medical research and patient care, with electronic health records (EHRs) containing a wealth of structured and unstructured data. Extracting valuable information from unstructured clinical text presents a significant challenge, necessitating automated tools for efficient data mining. Natural language processing (NLP) methods have been pivotal in this endeavor, aiming to extract crucial clinical concepts embedded within free-form text. Our research addresses the imperative for robust biomedical entity extraction, focusing specifically on inflammatory bowel disease (IBD). Leveraging novel domain-specific pre-training and entity-aware masking strategies with contrastive learning, we fine-tune and adapt a general language model to be better adapted to IBD-related information extraction scenarios. Our named entity recognition (NER) tool streamlines the retrieval process, supporting annotation, correction, and visualization functionalities. In summary, we developed a comprehensive pipeline for clinical Dutch NER encompassing an efficient domain adaptation strategy with domain-aware masking and model fine-tuning enhancements, and an end-to-end entity extraction tool, significantly advancing medical record curation and clinical workflows.

## 1 Introduction

The widespread adoption of electronic health records (EHRs), encompassing both structured, coded data and unstructured clinical text, has become integral to research in various clinical applications. Clinical reports, predominantly comprised of free-form unstructured text alongside structured patient data, pose a challenge in efficiently extracting valuable information. Automated tools for mining and extracting insights from this diverse data not only enhance communication among healthcare professionals but also contribute to improved patient care and a more thorough evaluation of healthcare outcomes, aligning with established standards of care. Beyond enhancing digital healthcare experiences, mining EHRs holds significant applications in advancing medical research.

A pivotal challenge in leveraging EHRs is the extraction of patient information embedded in the unstructured clinical text. Vital patient data, including family history, adverse drug effects, and social, behavioral, and environmental determinants of health, are often exclusively documented in this format. Consequently, significant efforts have been directed toward developing natural language processing (NLP) methods and tools to extract crucial clinical concepts from unstructured clinical text. For accurate medical decision-making, clinicians must review extensive medical documents covering therapeutic procedures, drug regimens, clinical trials, cohort selection, and interpretative assessments. However, manual review of unstructured clinical text is time and energy-intensive. Access to medical-related entities within the text enhances document readability and improves the effectiveness of clinical experts, providing them with essential information to identify key segments in a medical record.

The digitization of patient documents into EHRs has been a transformative trend in healthcare institutes, facilitating data management, patient history tracking, and research endeavors. These EHRs, comprised of rich information collected from patients, have attracted investments from insurance companies and pharmaceutical industries seeking to extract valuable medical information. Named entity recognition (NER) is a widely adopted approach for extracting medical information from unstructured clinical documents, with applications in pharmacovigilance, drug and disease NER, and other medical domains. Advanced NER approaches aim to automatically detect and classify medical entities into corresponding categories, thereby structuring medical text and enhancing the quality of medical services. The primary objective of our research is to empower medical practitioners, clinicians, nurses, and doctors with a highly accurate and efficient tool for swiftly retrieving relevant information from clinical reports. Clinical registries are valuable assets for hospitals. They contain important longitudinal medical information on clinical outcomes, allowing clinicians to improve their decision making and enabling clinical research. The aim of our research is to build a proof-of-concept system for biomedical entity extraction from clinical reports written in natural language. This system is specifically tailored to automatically extract various entity types related to inflammatory bowel disease (IBD). IBD refers to a group of chronic inflammatory conditions affecting the gastrointestinal tract, primarily including Crohn's disease and ulcerative colitis. These conditions are characterized by symptoms such as abdominal pain, diarrhea, weight loss, and fatigue. IBD is a complex disease with both genetic and environmental factors contributing to its pathogenesis. The variability in clinical presentation and the need for personalized treatment plans make it a challenging domain for clinical data mining, where automated extraction of relevant medical entities and information is crucial for improving clinical decision-making and patient outcomes. The choice of the IBD domain is motivated by the availability of patient reports that were already partially manually annotated with relevant named entities and because of the use of the extracted data in several past and ongoing clinical research projects. To build the proof-of-concept system we leverage domain and entity-aware pre-training strategies and fine-tune a general language model (LM) to cater to the nuances of the clinical domain. This adaptation ensures that the model excels in extracting relevant information related to the IBD domain.

Access to extracted medical entities within the text improves document readability, subsequently increasing the effectiveness of clinical experts. By providing essential information, our approach aids medical professionals in identifying key segments within a medical record efficiently. There is a growing interest in crafting specialized language models like BioBERT (Lee et al., 2020) or ClinicalBERT (Huang et al., 2019), tapping into the extensive medical literature. Yet, pre-training is costly, and conventional masked language models with random masking may not sufficiently adapt to specific domains. By directing the model to prioritize key domain-specific entities, we enhance its knowledge for better task performance. Additionally, employing contrastive learning helps create more distinct token representations during pre-training, yielding more transferable representations for improved performance during fine-tuning for the end task.

**Figure 1:**
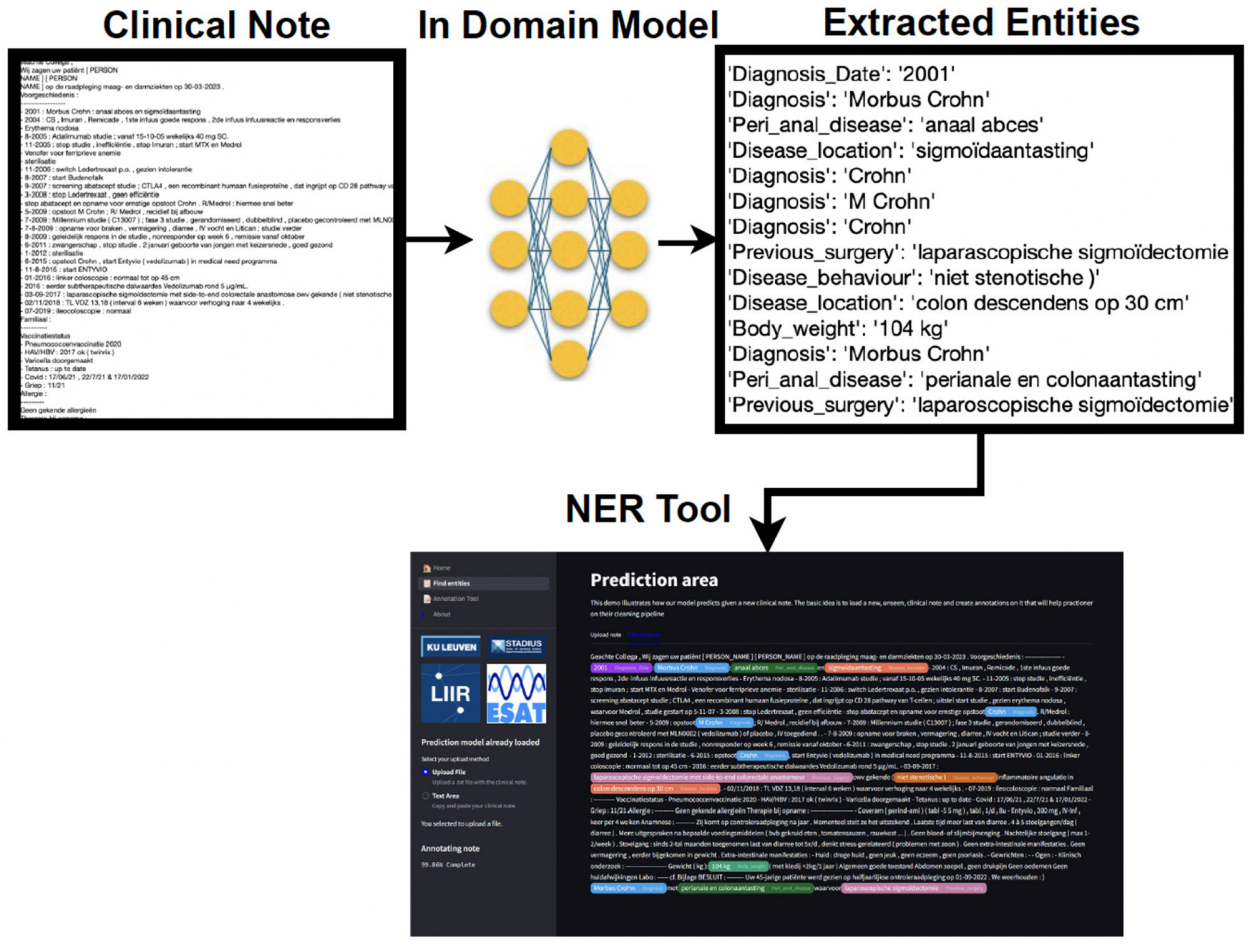
A simplified output of the clinical entity extraction pipeline. A preprocessed clinical note is used as input for our in-domain model. The latter finds the extracted entities, and those are highlighted in the NER tool, associating one color per entity. The practitioner is able to load, edit, and save the annotations that later on can be reused as (pseudo-)labeled data.

To operationalize our research, we have developed a NER tool (see Figure 1). This tool not only predicts entities in unseen new clinical notes but also offers options for annotating new notes or correcting existing annotations. Additionally, the tool provides a visualization of predictions alongside true annotations, displaying clinical notes with highlighted entities to improve document readability. Overall, our NER tool is designed to streamline the information retrieval process for medical practitioners, fostering a balance of accuracy and efficiency in clinical workflows.

In this paper, we make the following contributions:

1. Develop a comprehensive pipeline for clinical Dutch NER in the inflammatory bowel disease (IBD) domain, covering annotation, pre-training, fine-tuning, and the creation of an end-to-end clinical Dutch NER tool.
2. Adapt a general Dutch language model to the IBD domain through continued pre-training, introducing entity and domain-aware masking strategies using contrastive learning during continued pre-training to enhance task and domain adaptation.
3. Use iterative masking with pseudo-labels after an initial fine-tuning to bring the pre-trained model closer to the end task. Masking based on differences in vocabulary and context provides robust representations for continued pre-training.
4. Improve model fine-tuning through additional information fusion and consistency training, enhancing overall model performance. We also experimented with a multitask setting to simultaneously learn both sentence-level categories and NER tags given an input sentence to deal with low-performing entity classes.
5. An end-to-end entity extraction pipeline tool that enhances medical record curation, merging annotation, and prediction capabilities by integrating the NER model functionalities into a single platform marks a significant step forward compared to existing solutions.

## 2 Related work

Named entity recognition (NER), which involves extracting and categorizing entities from text, is a fundamental task in natural language processing, pivotal across diverse domains like medical coding, financial analysis (Francis et al., 2019), and legal document parsing. Typically, addressing specific NER challenges for distinct domain-specific entity types necessitates custom model creation. Over the past 5 years, the conventional approach to building such models has relied on utilizing transformer encoders pre-trained via self-supervised learning to fulfill masked language modeling (MLM) objectives, exemplified by models like those in the BERT family (Devlin et al., 2019; Huang et al., 2019; Lee et al., 2020). Subsequently, these foundational models undergo supervised fine-tuning on human-annotated data, employing various techniques for supervised NER tasks. These techniques can be classified based on their formulation of the task, spanning sequence labeling (Chiu and Nichols, 2015; Katiyar and Cardie, 2018), parsing (Yu et al., 2020), span classification (Fu et al., 2021), sequence-to-sequence approaches (Yan et al., 2021), and machine reading comprehension (Xue et al., 2020). In our work, we focus on the clinical entity extraction task tailored to the IBD domain and model it as a sequence labeling problem. We additionally inject section title information from the clinical note into the NER model to additionally infuse structured information into the model thereby demonstrating the importance of augmenting the context in which certain entities occur.

Recent studies [BioBERT (Lee et al., 2020), TAPT (Gururangan et al., 2020), and ClinicalBERT (Huang et al., 2019)] have demonstrated the efficacy of further adapting a general pre-trained model through continued pre-training on a more relevant set of downstream tasks. Task adaptive pre-training (TAPT) involves pre-training on the specific end-task and dataset itself, leveraging unsupervised objectives. This method enhances model performance for the target task while being computationally efficient (Francis and Moens, 2022a,b; Gururangan et al., 2020). Although pre-training learns representations for all words using self-supervised tasks, not all tokens are equally important for downstream fine-tuning tasks (Zhou et al., 2022b; Pergola et al., 2021). Many pre-trained words remain unused in fine-tuning, while crucial words for the task may lack proper representation due to limited labeled data.

Masking plays a pivotal role in self-supervised learning (SSL), where models strive to reconstruct hidden portions of data based on contextual cues. Its impact has been profound across various domains, including language (Devlin et al., 2019), vision (Li et al., 2021), and speech (Hsu et al., 2021), leading to breakthroughs in performance on diverse tasks. These transformer-based models typically pre-trained through self-supervised masked language model [MLM (Devlin et al., 2019)] objectives, predict randomly masked subsets of input tokens. Traditional approaches, such as static masking in BERT, have been extended by dynamic and adaptive masking strategies that tailor masking patterns based on context or task-specific requirements. Recent studies have explored entity-aware masking, where knowledge of semantic roles or external ontologies guides the selection of masked entities (Zhou et al., 2022b; Pergola et al., 2021), mask-guided BERT for few-shot text classification (Liao et al., 2024) and masking network parameters (Zheng et al., 2023). Other research directions include adversarial masking techniques, which create challenging training examples by masking tokens that maximize model uncertainty, thereby improving downstream performance. While previous studies have focused on the quantity of masked data and mostly resort to random masking or span/phrase masking approaches, the decision of what to mask to adapt a model to the specific task domain integrating contrastive learning remains understudied in SSL, although there have been works to integrate external knowledge (Sun et al., 2020).

In clinical NLP tasks, which often revolve around entities such as clinical named entity recognition, clinical negation extraction, and clinical relation discovery, existing masking strategies during pre-training have not adequately focused on clinical entities. Our paper uses a methodology to produce a model specifically focused on clinical entities. We continue pre-training a Dutch model on an IBD clinical corpus, along with a novel entity-centric masking strategy with contrastive learning to integrate domain knowledge into the learning process. Contrastive learning has become a cornerstone for representation learning across modalities, with frameworks like SimCLR (Chen et al., 2020) and CLIP (Radford et al., 2021) demonstrating the power of contrastive objectives in pre-training. In NLP, contrastive methods have been adapted for tasks such as sentence similarity, retrieval, and knowledge base completion. Building on this foundation, our work introduces a task-specific contrastive learning framework that incorporates domain-specific entity masking to address domain imbalances by dynamically adjusting the contrastive loss to emphasize even the rare or under-represented entities. This approach demonstrates superior generalization, especially in data-scarce scenarios. In this paper, we argue that the key to obtaining more discriminative and transferrable representations lies in learning contrastive token-level representations using entity-specific masking during pre-training. We evaluate our entity-centric masking strategy on the IBD entity extraction task.

Techniques from NLP have shown significant promise in fields like cancer research and genetic disease diagnosis. In oncology, contrastive methods have been applied to histopathology image analysis, aiding in tumor classification, and biomarker discovery (Ciga et al., 2021). Similarly, in genomics, adaptive masking has improved variant detection and gene-phenotype mapping by leveraging domain-specific embeddings (Huang et al., 2019). These cross-domain adaptations underscore the versatility of these techniques in handling complex, high-dimensional data. Our model's adaptable design makes it well-suited to extend for such applications, offering a framework that can seamlessly integrate into domains requiring robust entity representation.

Clinical annotation tools are designed to organize and interpret complex medical data, enhancing research and patient care through efficient data categorization and insight extraction. Most annotation tools focus only on manual annotation of existing clinical records (for example, Klie et al., 2018, Islamaj et al., 2020, and Han et al., 2021). Moreover, our primary focus relies not only on the annotation phase but also on the prediction phase, as it may enhance the annotation process if predictions are reliable. Tools that provide machine learning support have different limitations, such as compatibility with modern machine learning frameworks (INCEpTION Klie et al., 2018) and being able to be used without paying a costly license (Prodi.gy). Therefore, our tool is designed so that the medical team can upload any pre-trained model for the creation of pseudo-labels and, in the same pipeline, update the entities being created so the expert can decide whether to use, delete, or change them.

## 3 Methods

### 3.1 Entity extraction pipeline

**Figure 2:**
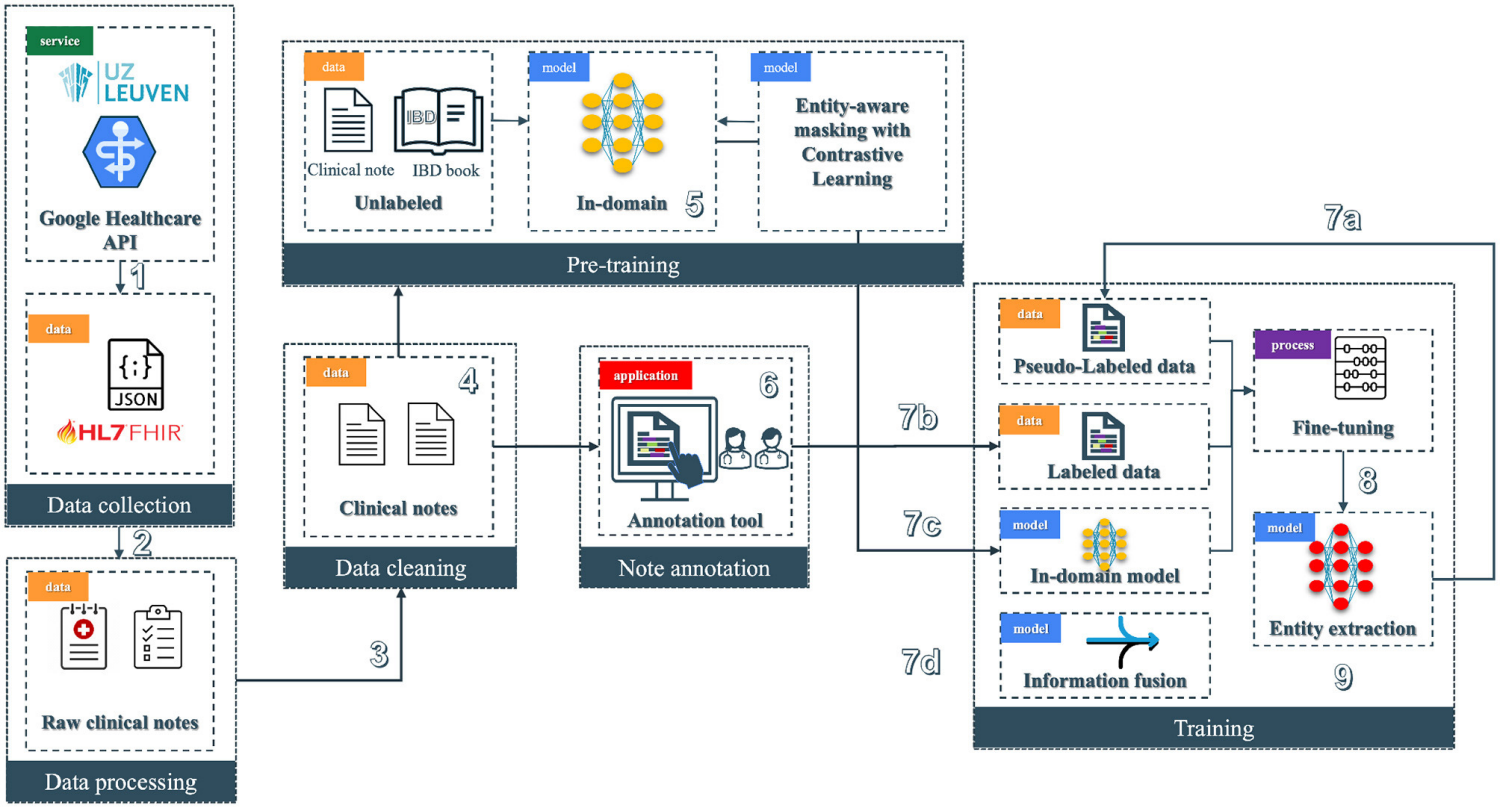
Overview of the clinical entity extraction pipeline. The workflow begins with Data Collection (1), where raw clinical notes and JSON-based HL7 FHIR data are retrieved from a Google Healthcare API provided by UZ Leuven, followed by Data Processing (2) to prepare raw notes for downstream tasks. Next, Data Cleaning (3) removes noise from clinical notes, enabling the Pre-training phase (4), where an in-domain model (5) is trained on unlabeled data using contrastive learning with entity-aware masking. Annotated clinical notes are generated through a specialized Annotation Tool (6) to produce labeled data. In the Training phase (7), labeled, pseudo-labeled, and fine-tuned data streams are integrated alongside pre-trained in-domain models: 7a involves the usage of pseudo-labeled data, 7b incorporates the labeled data from the annotation tool, 7c involves the in-domain models, and 7d focuses on information fusion. Finally, the pipeline finishes with the entity extraction model (8), where the fine-tuned model is deployed (9) to extract entities from clinical text.

The pipeline (see Figure 2) of the NER system for clinical text is divided into six modules; among those, we have data collection, processing, cleaning, note annotation, pre-training, and training. Each step integrates tools and models to refine the dataset and to improve the accuracy of NER tasks in a healthcare context.

#### 3.1.1 Data preprocessing

The original data store is in a FHIR (Fast Healthcare Interoperability Resources) server located at UZ Leuven premises (see Figure 2 block 1). FHIR is a standard for exchanging healthcare information electronically.

The training data is annotated by domain experts in IBD using the BRAT annotation tool (Stenetorp et al., 2012). The first step consists of pre-processing the text to convert it from the BRAT format to make it suitable for the transformer-based model. Further, we cleaned and split the clinical text into sentences, and we added special tokens at the beginning and end of the sentence. The [CLS] token is used for indicating the beginning of the sentence, [SEP] for indicating its end, and [PAD] tokens are added to make the input length uniform. These special tokens vary according to the models used. Our model employs the BIO tagging annotation schema that identifies tokens in the text with specific tags: (i) Beginning (B) of the entity, (ii) Inside (I) of the entity, and (iii) Outside (O) entities. The “O” tag signifies text that is not relevant to any entity category.

Since there is a substantial mismatch between the number of records belonging to each entity category we use sampling strategies like up-sampling.

#### 3.1.2 Domain-adaptive pre-training

Traditional open-domain language models (LM), like the Transformer (Vaswani et al., 2017) and its variants (Devlin et al., 2019; Lee et al., 2020; Liu et al., 2019), are further pre-trained on in-domain corpora to extract domain knowledge and enhance performance on domain-specific downstream applications.

To incorporate in-domain knowledge, we leverage masked-language modeling (MLM) to continually pre-train the Dutch LM (Delobelle et al., 2020). For an input sentence *X* = *x*_1_, *x*_2_, ...., *x*_*n*_, we first tokenize the sentence using a pre-trained Dutch Roberta-based tokenizer, generating *n* tokens. The MLM mask function masks *p%* of the tokens, creating a mask vector to filter out masked tokens. The value of *p* is usually set to 0.15 in a standard MLM masking task (Devlin et al., 2019). The masked sequence is fed into the model, denoted as a function *F*. We utilize the cross-entropy (CE) loss function to compute the MLM loss for sentence *x*_*i*_. Once the MLM loss curve converges, domain-specific knowledge from unlabeled clinical notes and inflammatory bowel disease (IBD) specific textbook data is encoded into the model's parameters. We save the model's weights for downstream applications, such as clinical NER on annotated datasets. Further, we modify the simple MLM objective of the LM to include entity-specific objective functions incorporating contrastive learning to learn a better domain-adapted representation for downstream clinical tasks. Entity-specific pre-training strategies are detailed in Sections 3.2, 3.3.

#### 3.1.3 Model fine-tuning

We fine-tune the above domain-adapted clinical Dutch LM model to improve its ability to learn accurate representations of target domain entities, particularly clinical entities. During fine-tuning, we adjust the weights of the pre-trained model for the initialization of the NER task. The embeddings generated by the transformer-based model are used in a token-level classification task, with a linear classification layer added atop the encoder stack to generate a prediction matrix for the input sequence. This is used to predict the NER tags based on clinical concepts present in the training corpora of the target domain (annotated training corpora of IBD clinical notes). Following the fine-tuning process, we evaluate the effectiveness of the model in entity extraction by assessing its performance on clinical entity extraction tasks. To evaluate the NER capabilities of our fine-tuned model, we prepare a separate dataset comprising annotated clinical notes unseen during training. This evaluation dataset encompasses a diverse set of examples, covering various clinical entities. Carefully curated, this dataset enables a comprehensive assessment of the model's generalization across different scenarios.

### 3.2 Entity MLM

To further enhance our initial pre-trained language model configuration, we incorporate entity masking during task-specific finetuning. This technique extends the pre-trained masked language model (MLM) objective by refining it through additional training on masked sentences (Zhou et al., 2022b). Here, only entity/concept tokens are randomly masked as opposed to general MLM masking. This involves inserting a label token both before and after each entity token, treating these added labels as ordinary context tokens during MLM. Consequently, predicting the masked entity token becomes dependent not just on its surrounding context but also on its designated label. Label tokens are inserted around the masked concepts which act as a form of context labels to direct the model prediction.

**Figure 3:**
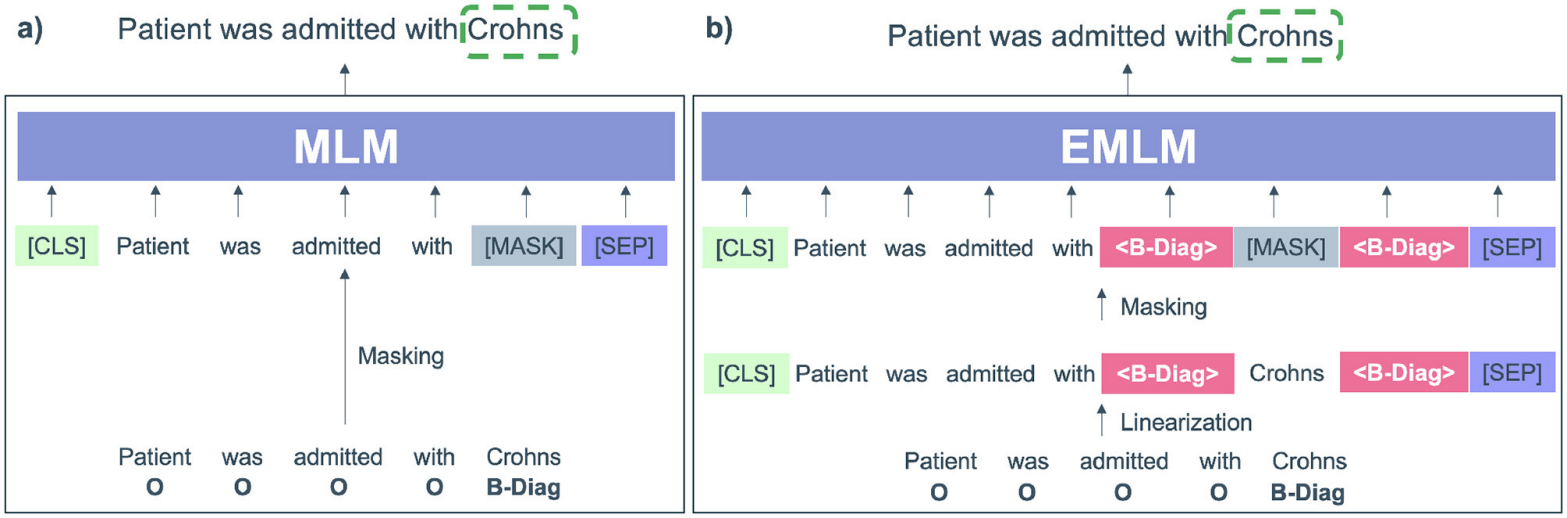
**(A)** Input modified by the pre-trained masked language model (MLM) where only the entity (Crohns) is masked. **(B)** Input modified for Entity-specific Masking (EMLM) were < B-diag> labels referring to diagnosis are added before and after the entity token Crohns.

By injecting label information (see Figure 3) and fine-tuning the label-enhanced data, the entity MLM (EMLM) leverages rich pre-training knowledge to enhance entity diversity while significantly reducing token-label misalignment during fine-tuning. This is achieved by adding a label token before and after each entity token, treating them as regular context tokens. The resulting modified sequence is then used for further fine-tuning of the masked entity model, ensuring that its prediction is influenced by the inserted label tokens. Notably, the embeddings of label tokens are initialized with those of tokens semantically related to the label names, enhancing the semantic coherence of the modified input sequence and reducing the difficulty of fine-tuning new sequences.

By injecting label information and finetuning the label-enhanced data, the entity MLM (EMLM) leverages rich prior pre-training knowledge into the model parameters. The resulting modified sequence is then used for further finetuning of the masked entity model, ensuring that its prediction is influenced by the inserted label tokens. Notably, the embeddings of label tokens are initialized with those of tokens semantically related to the label names, enhancing the semantic coherence of the modified input sequence and reducing the difficulty of finetuning new sequences.

In contrast to MLM, where any token can be randomly masked, clinical entity masking specifically targets concept entity tokens during finetuning. At the beginning of each finetuning epoch, entity tokens within the input sentence with inserted labels, denoted as *X*, are selectively masked at random according to a masking ratio η. The entity masking model is subsequently trained on this corrupted sentence *X*^*m*^ to optimize the probabilities of masked entity tokens and reconstruct the original input sequence *X*.


(1) $$\begin{array}{l} \underset{\theta }{\max}\log P\left(X|X^{m};\theta \right)\approx \overset{n}{\underset{i=1}{\sum }}m_{i}\log P\left(x_{i}|X^{m};\theta \right) \end{array}$$

where θ represents the parameters of EMLM, *n* is the number of tokens in *X*^*m*^, *x*_*i*_ is the original token in *X*, *m*_*i*_ = 1 if *x*_*i*_ is masked and otherwise *m*_*i*_ = 0.

This results in the following negative log-likelihood loss.


(2) $$\begin{array}{l} L_{\text{E-MLM}}=-\overset{n}{\underset{i=1}{\sum }}\log P\left(x_{i}|X^{m};\theta \right) \end{array}$$

Here, the model learns to reconstruct the original sequence *X* by predicting each masked entity token *x*_*i*_, given the surrounding context *X*^*m*^ and label tokens. Through this fine-tuning process, the model learns to effectively utilize both context and label information for predicting masked entity tokens. In this phase, rather than randomly choosing the tokens to be masked, we inform the model of the relevant tokens to pay attention to and encourage the model to refine its representations using the new surrounding context which includes the label information as well.

The use of label tokens enriches the context surrounding masked entities, making the predictions more robust and contextually informed. Unlike generic MLM, which masks random tokens, EMLM focuses on semantically meaningful tokens (entities), helping the model to better capture domain-specific relationships. By including labels, the model reduces ambiguity and leverages prior semantic knowledge, improving fine-tuning efficiency for downstream tasks.

### 3.3 Entity-aware contrastive learning

To further enhance and augment the representation learning capability of entity masking models, contrastive learning is employed to refine the representations of masked entity tokens by aligning them with their unmasked counterparts while separating them from representations of other tokens. We employ a dual-model approach, consisting of a student model referred to as *S* and a teacher model denoted as *T*, both initialized from the identical pre-trained Dutch Roberta model architecture. During the training process, we maintain the teacher model's layers in a fixed state and concentrate solely on refining the parameters of the student model *S*.

Given an input sequence *X* = [*x*_1_, ..., *x*_*n*_], we incorporate entity labels, masking the corresponding entities according to the methodology outlined in the Section 3.2. Subsequently, this masked sequence *X*^*m*^, is fed into the student model, generating a contextual representation $h^{m}=\left[h_{1}^{m},...,h_{n}^{m}\right]$. Simultaneously, the teacher model is fed the original sequence *x* as input, yielding a representation *h* = [*h*_1_, ..., *h*_*n*_]. For each masked entity token $x_{i}^{m}$, its representation $h_{i}^{m}$ (from the student) is encouraged to align closely with the corresponding ground-truth token representation *h*_*i*_ (from the teacher). The masked entity-aware contrastive learning objective $L_{CL}$ is then defined as


(3) $$\begin{array}{l} L_{CL}=-\overset{n}{\underset{i=1}{\sum }}x_{i}^{m}\log\frac{\exp\left(\mathrm{sim}\left(h_{i}^{m},h_{i}\right)/\tau \right)}{\overset{n}{\underset{j=1}{\sum }}\exp\left(\mathrm{sim}\left(h_{i}^{m},h_{j}\right)/\tau \right)} \end{array}$$

where $x_{i}^{m}=1$ if $x_{i}^{m}$ is a masked clinical entity token, otherwise $x_{i}^{m}=0$. τ is a temperature hyper-parameter that controls the sensitivity of the model to differences between positive and negative pairs and is selected through hyperparameter tuning and *sim*(., .) computes the cosine similarity. The contrastive loss promotes the similarity between the representation of the reconstructed masked token $h_{i}^{m}$ and the representation of its ground truth token *h*_*i*_ in the latent representation space. Through the softmax normalization the representation of the reconstructed masked token $h_{i}^{m}$ will become more distinct from the representations of other tokens *h*_*j*_.

The underlying concept is for the student to refine its representation of a masked entity token to closely align with the “reference” representation provided by the teacher while distancing itself from other tokens within the sequence. This fosters the learning of token representations by the student that are more discriminative and consequently, better suited to follow an isotropic distribution (Su et al., 2022). In the context of representation learning, having token representations that follow an isotropic distribution implies that each token's representation is equally informative and contributes consistently to the overall understanding of the input sequence (Ethayarajh, 2019). This uniformity can facilitate more effective downstream tasks, as the representations are well-distributed and capture the relevant information in a balanced manner.

To drive this learning process, we employ EMLM and contrastive learning (CL) objectives. EMLM ensures that the model accurately predicts masked entities based on context and label information. Contrastive Learning refines the token embeddings, making them more robust, discriminative, and isotropically distributed. Together, these objectives allow the model to leverage entity-specific information effectively and produce representations better suited for downstream tasks. The overall learning objective $L_{pretrain}$ for the student model during the continual pre-training phase is expressed as:


(4) $$\begin{array}{l} L_{pretrain}=L_{CL}+L_{E-MLM} \end{array}$$

Upon completion of this learning phase, we proceed to fine-tune the student model for downstream tasks.

### 3.4 Fine-tuning entity extraction model

During the fine-tuning step, we model entity extraction as a sequence labeling task such that given an input sentence of *n* words *X* = [*x*_1_, *x*_2_, ..., *x*_*n*_], the output is a sequence of named entity labels *Y* = [*y*_1_, *y*_2_, ..., *y*_*n*_]. The named entity labels are in the form of BIO labels assigned at the token level. For each *x*_*i*_, the goal is to predict the corresponding label *y*_*i*_. The input sentence *X* is fed into the encoder [pre-trained Dutch-based language model (Delobelle et al., 2020)] to obtain the hidden representation. Further, this is fed into a fully connected layer followed by a SoftMax layer. The goal is to classify each token in *X* and assign it to a corresponding label *y*∈*C*, where *C* is a predefined list of all label types in the IBD domain (e.g., diagnosis, disease location, disease behavior, etc) in BIO format. The loss function is the cross-entropy function with *y*_*i*_ as the true label for the *i*-th example and ŷ_*i*_ as the predicted probability distribution over labels for the *i*-th example. For the dataset with *N* examples, the overall objective function is computed as the average of the losses over all examples.


(5) $$\begin{array}{l} L_{CE}=-\frac{1}{N}\overset{N}{\underset{i=1}{\sum }}\overset{C}{\underset{j=1}{\sum }}y_{i,j}\log\left({ŷ}_{i,j}\right) \end{array}$$

NER label predictions are dependent on predictions of surrounding words. It has been shown that structured prediction models can improve NER performance, such as conditional random fields (CRF). A CRF is a type of probabilistic model used for structured prediction tasks, such as sequence labeling or segmentation. It models the conditional probability distribution of label sequences given input observations. Here we simulate a CRF in the neural network by an additional layer for modeling NER label dependencies as weights. The following sections describe how we enhance the fine-tuning performance of the entity extraction model.

#### 3.4.1 Pseudo label and Rdrop regularization

To further augment the training data used for training the entity extraction model, we use semi-supervised learning (SSL) and gather a portion of raw, unlabeled clinical reports and construct them as text files denoted as T with N sentences. SSL involves propagating label information from a small amount of labeled data to a large number of unlabelled samples. We leverage the continually pre-trained in-domain Dutch Roberta model to generate predictions for the unlabelled clinical notes. These generated predictions serve as pseudo-labels for the subsequent iteration of model training. Pseudo labels increase the training dataset even though it can only be considered as silver standard data without a human-in-the-loop setup.

In this approach, we also integrate Rdrop (Liang et al., 2021) consistency training. By regulating multiple predictions on varied views of the same data point, consistency training reduces sensitivity to noisy labels. This enhances the model's robustness to dropout-induced perturbations, thereby mitigating overfitting and promoting better generalization. Recent works have applied consistency training on NER (Zhou et al., 2022a; Francis and Moens, 2023b), including token-level and sequence-level consistency methods. Dropout acts as a form of perturbation ϕ at the representational level. By regularizing the model to be invariant to different random dropouts, we encourage the model to make predictions based on more diverse features rather than overfitting certain spurious features.

Concretely, we pass the same sentence *X* through the encoder twice. As a result of different stochastic dropouts in each pass, we obtain two different sets of token representations for *X*. Subsequently, the model outputs two different token-level probability distributions *P*_1_(*y*_*i*_|*x*_*i*_) and *P*_2_(*y*_*i*_|*x*_*i*_) over the label space *Y*. Subsequently, bidirectional KL divergence is employed to compute the dropout-based consistency loss together with the cross-entropy loss.


(6) $$\begin{array}{l} {ℒ}_{rdrop}=\frac{1}{n}\sum_{x_{i}\in X}\frac{1}{2}[KL(P_{1}\left(y_{i}|x_{i}\right)\|P_{2}\left(y_{i}|x_{i}\right)\\ \;+\mathrm{KL}\left(P_{2}\left(y_{i}|x_{i}\right)\|P_{1}\left(y_{i}|x_{i}\right)\right)] \end{array}$$

These Rdrop based consistency loss is combined with the supervised cross-entropy loss ($L_{CE}$) to form the combined training objective ($L_{finetune}$) where α is the weight cofficient.


(7) $$\begin{array}{l} L_{finetune}=L_{CE}+\alpha L_{rdrop} \end{array}$$

#### 3.4.2 Section information fusion

To enhance the contextual representation of the NER model, we utilize the section labels obtained from the clinical note as additional attributes to fuse into NER. Section headings can be seen as structured data which are rich additional information to reduce classification errors. We can inject these attribute representations as additional information at multiple layers of the model (Francis and Moens, 2023a). In each layer of the model, we see some form of non-linear functions incorporated, using the general equation *g*(*f*(*x*)) = *g*(*Wx*+*b*), where *f*(*x*) refers to a transformation function of *x, g* is a non-linear activation function, *W* and *b* are weight matrix and bias, respectively. We can represent the attributes as the bias *b* to one of these locations by modifying them to accept section title label information (*s*) as inputs, i.e., *f*(*x, s*).

Injecting attributes to the word embedding means that we bias the probability of a word belonging to a particular class label (based on the section label). We modify the Dutch model to add extra attributes as input in addition to the tokens. Here we allow the model to process additional information on the token level. This is achieved by having an extra embedding layer for the extra attribute. The full embedding vector representation of an input token is then obtained by concatenating the embeddings of the token and the additional attribute added. Injecting attributes to the text means that we concat the section label information along with input text as a piece of additional context information. By injecting the additional attributes into the NER classifier, we bias the probability distribution of the NER classifier based on the final encoded hidden representation. When we represent the section title labels as a bias in the NER classifier, this produces a biased logit vector that classifies a token as a particular entity by shifting the final probability distribution of the label class.

**Figure 4:**
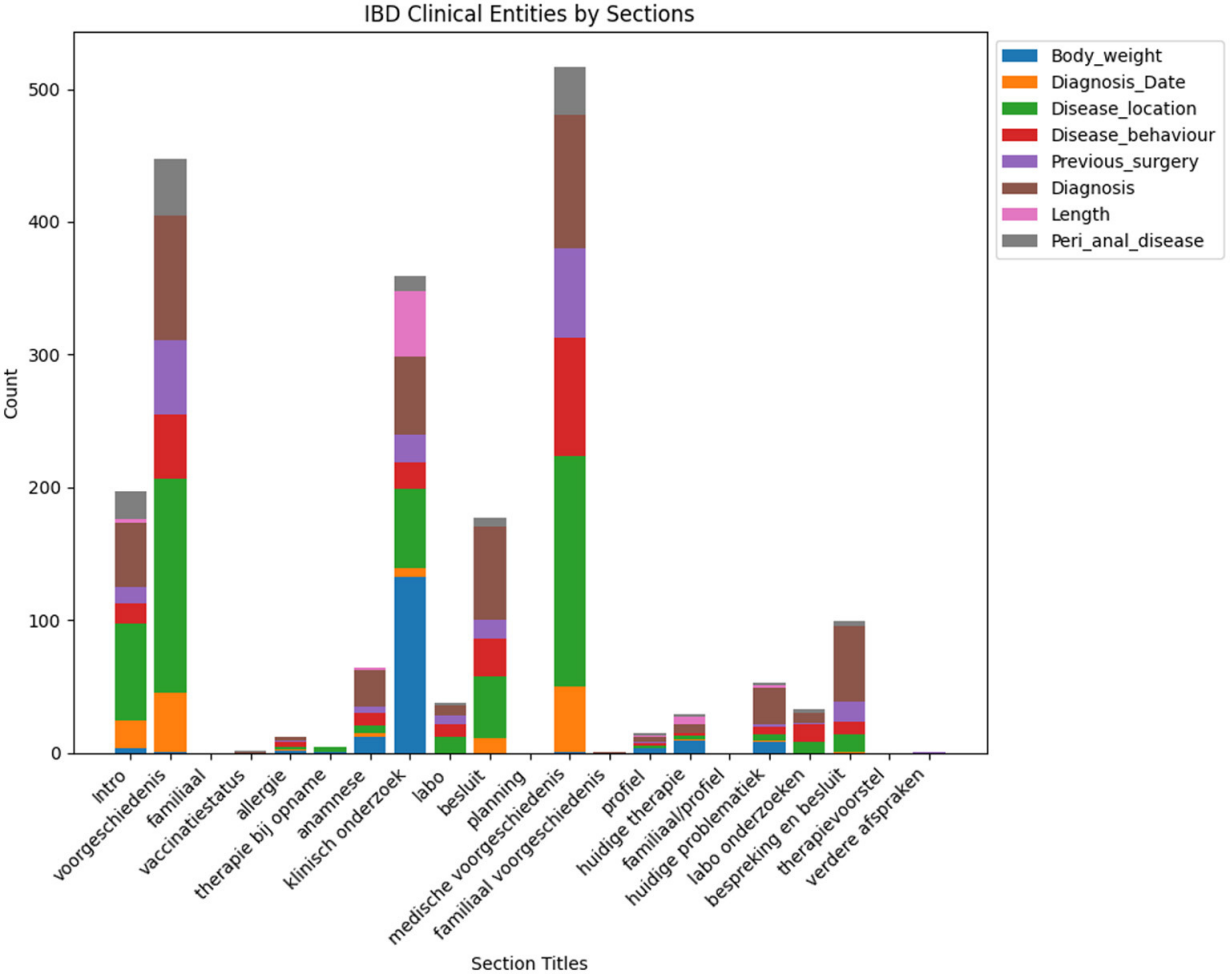
This figure illustrates the frequency of different clinical entities categorized under each section title. It provides a comparative analysis of the occurrence count for each entity across common clinical sections found in clinical notes.

Incorporating section title (see Figure 4) information into the NER model helps the model to bias the recognition of certain entities based on the context of section title information. Certain entities can fall under certain sections whereas other entities cannot be present in a section. For example, the diagnosis entity in the family history section of the clinical report is not a correct entity recognition.

#### 3.4.3 Multi-task setting to boost low performing entity labels

With multi-task learning (MTL), a model is trained to perform multiple similar tasks simultaneously such that learning from multiple tasks can lead to improved generalization and overall performance. The goal is to simultaneously learn to predict if an input sentence has peri_anal disease mentioned (least performing entity) and also detect NER tags within the sentence. By jointly learning from related tasks, the model can leverage commonalities and dependencies, leading to better performance compared to training separate models for each task.

The overall framework consists of three components, namely (1) training with supervised cross-entropy loss (NER labels), (2) binary cross-entropy loss (sentence classification), and (3) dropout-based consistency training (Rdrop).

To simultaneously learn both sentence-level categories and NER tags given an input sentence *x*, the Dutch model can be easily extended to a joint label classification and NER model. Depending on the hidden state of the [CLS] token (special token placed at the beginning), denoted *h*_1_, the sentence label is predicted as *y*_*i*_ = *SoftMax*(*W*_*i*_*h*_1_+*b*_*i*_). For NER, we feed the final hidden states of the other tokens *h*_2_, ..., *h*_*T*_ into a SoftMax layer to classify with the NER labels *y*_*s*_. The learning objective is to maximize the conditional probability *p*(*y*_*i*_, *y*_*s*_|*x*). The model is finetuned end-to-end by minimizing the cross-entropy loss. Here we simulate a conditional random field (CRF) (Vaswani et al., 2017) for modeling NER label dependencies, on top of the joint Dutch Roberta model.

Learning multiple tasks simultaneously can act as a form of regularization. The model is encouraged to discover robust and generalizable features that are useful across all tasks, which can help prevent overfitting and enhance the model's ability to generalize to new, unseen data.

## 4 End-to-end prototype tool

**Figure 5:**
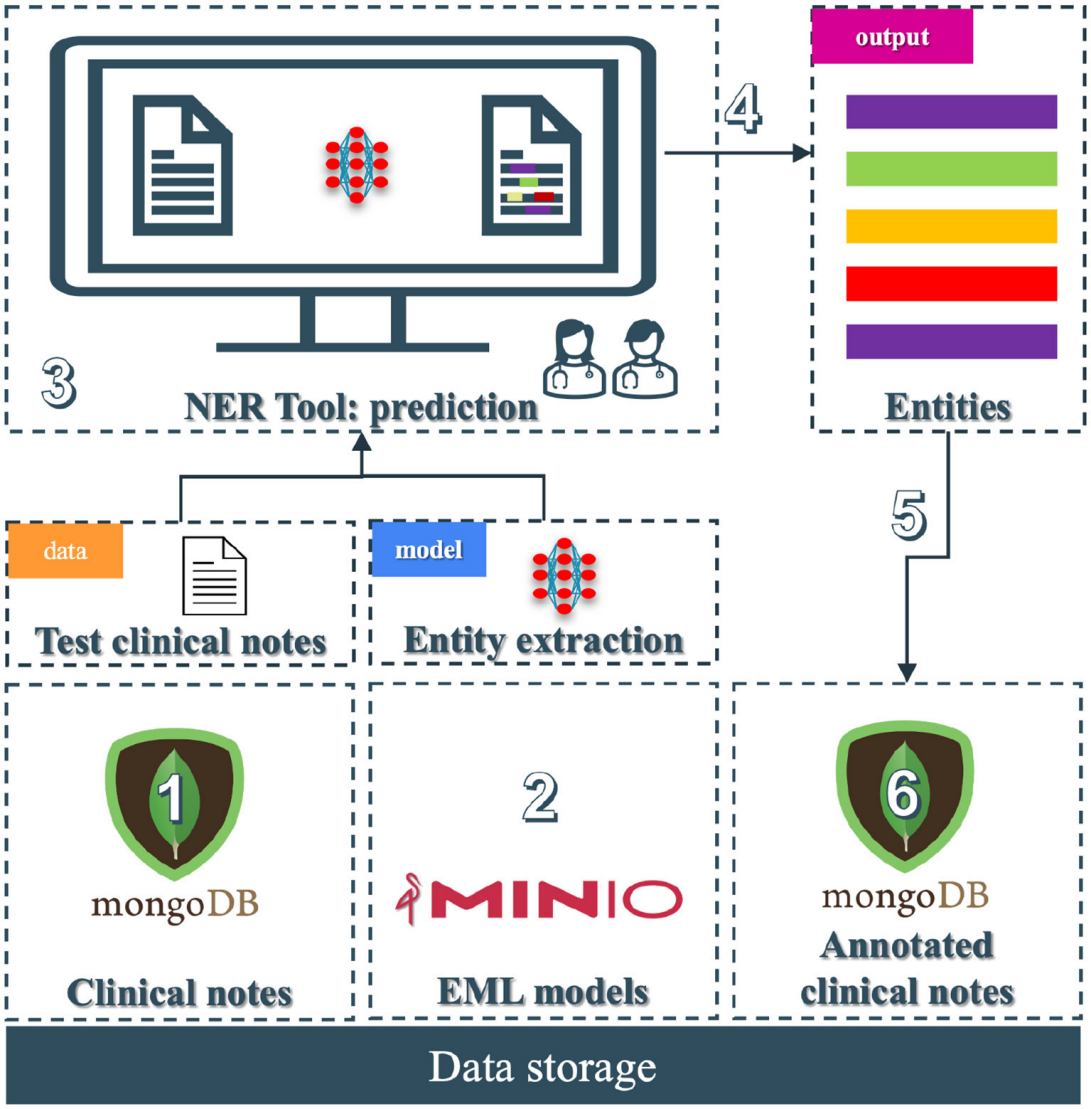
End-to-end prototype tool: (1) Unannotated clinical notes that the practitioners can add securely into a MongoDB collection; (2) The NER models stored in a MiniO object storage; (3) The NER tool where practitioners load unannotated clinical notes and perform the prediction task to store the entities; (4) The entities are being predicted and displayed; (5) The medical team can edit, transform, or add the predictions; and (6) store them in another MongoDB collection.

An end-to-end entity extraction pipeline tool to enhance medical record curation was developed (see Figure 5). Using pre-trained models, it aims to improve usability and efficiency by combining features from known annotation tools and a named-entity recognition (NER) pipeline. The tool enables users to easily manage annotations–adding, editing, visualizing, and predicting them through a user-friendly interface. It allows for retrieving clinical notes from a MongoDB (Inc., 2009) database and loads from a MiniO instance the NER model to facilitate annotation creation and efficient modification. Integrating annotation and prediction capabilities functionalities into a single platform marks a significant step forward in existing solutions.

This section outlines the development of a novel annotation tool, leveraging the Python-based framework Streamlit for its ease of use for creating web applications that enabled the implementation of a tool that facilitates the annotation of textual data, introduces functionalities critical for a secure and productive annotation environment and more importantly leverages on our NER models to increase the productivity of the medical team. In summary, this tool facilitates the annotation of clinical notes as most standard open-source options but enhances it using machine learning.

### 4.1 User authentication

A foundational aspect of our tool is incorporating a robust authentication system. Recognizing the sensitive nature of clinical notes, we have integrated mechanisms to create distinct user roles, ranging from administrators to annotators, ensuring that access is appropriately managed. Among the functionalities, we have:

- A secure authentication system allows for the creation of different user profiles and allows for controlled access based on customizable user roles in the configuration phase.
- Passwords are securely stored using industry-standard hashing techniques to ensure data privacy.

### 4.2 Data storage

MongoDB was chosen first for its flexibility and performance in handling small to medium-sized volumes of unstructured data to store clinical notes and second because of its compatibility with the FHIR resources, which are returned in JSON format. This NoSQL database allows for efficient retrieval and storage of clinical notes. Among the functionalities, we have:

- Retrieval given an ID of the clinical notes.
- Retrieval given a portion of the content of the clinical notes by using regular expressions.
- Storage of the clinical annotations distinguishing between predictions and actual annotations made by the medical team.
- Storage of custom non-existent clinical notes that can be uploaded either as a document or as text in a text-view.

### 4.3 Pretrained models for annotation

Central to our tool's functionality is the capability to make predictions using a pre-trained model in PyTorch while being stored in a MiniO (MinIO, 2016) instance. We decided using MiniO as it is a scalable and secure storage solution, making it ideal for handling large PyTorch model files efficiently enabling smooth upload, download, and version control in the case the tool is migrated to a cloud-based solution. Its compatibility with AWS S3 ensures seamless integration with PyTorch workflows. Being open-source and lightweight, MiniO offers a cost-effective and easy-to-deploy option for both on-premises and cloud environments. These choices not only capitalize on PyTorch's powerful machine-learning capabilities but also maintain the flexibility to incorporate models from other frameworks if needed. Therefore, the tool is framework-agnostic, allowing future integration with other popular libraries like TensorFlow, Keras, or scikit-learn. The pre-trained model automates the initial annotation process, generating pseudo-labels that can significantly accelerate the annotation workflow.

### 4.4 Interfaces

#### 4.4.1 Annotation view

**Figure 6:**
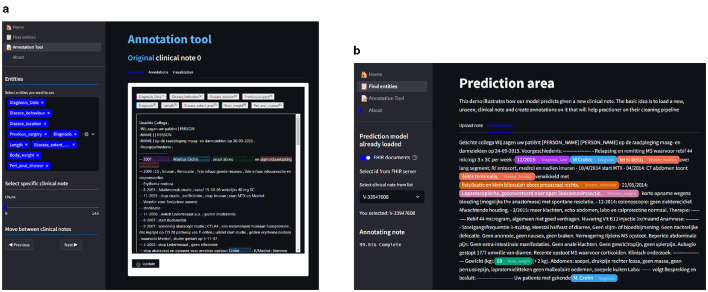
In **(A)**, we see the annotation view where we can add, edit, and save entities in the clinical notes. In **(B)**, we have the prediction view where we can select clinical notes from the database or upload custom clinical notes; after the upload, we can predict using the Clinical NER model to highlight the entities. Finally, the notes can be saved and uploaded to the database, where we can then edit the annotations on the annotation view. **(A)** Annotation view of the NER tool. **(B)** Prediction view of the NER tool.

The annotation view (Figure 6A) is a core feature designed for ease of use and efficiency. Users can edit, select, and save annotations directly within the interface. These annotations are then stored in MongoDB, allowing seamless integration between the annotation process and the data storage. This design facilitates a dynamic and flexible workflow, accommodating various annotation needs. Among the functionalities, we have:

- Configuration of desired entities by adding names and colors in a easy to understand configuration file.
- Intuitive, self-explained interface leveraging Label Studio annotation functionality without complex capabilities.

#### 4.4.2 Prediction view

The prediction view (Figure 6B) is the core feature of our tool, which uses the pre-trained model to predict pseudo-labels for previously unannotated clinical notes. This predictive functionality not only aids in streamlining the annotation process but also ensures a more comprehensive and accurate dataset, ready for further analysis and model training.

## 5 Data and experiments

The annotated dataset refers to the dataset of anonymized clinical notes that were manually labeled by clinical experts. The annotated training dataset comprises a total of 1, 711 clinical notes provided by the inflammatory bowel disease (IBD) department of UZ Leuven Hospital. The dataset was collected in 3 phases and annotated by three domain experts. The first phase consisted of intra-patient level notes while phases 2 and 3 consisted of inter-patient level notes. To ensure that the model generalizes to new patients, the test set is formed from phase 2 and phase 3 annotated clinical data. Annotation guidelines and training were provided to domain experts for the purpose of annotating relevant IBD clinical entities. To fine-tune transformer-based models for clinical entity extraction, we split annotated clinical notes into a training set (1, 463 clinical notes), a validation set (290 clinical notes), and a test set (250 clinical notes).

**Figure 7:**
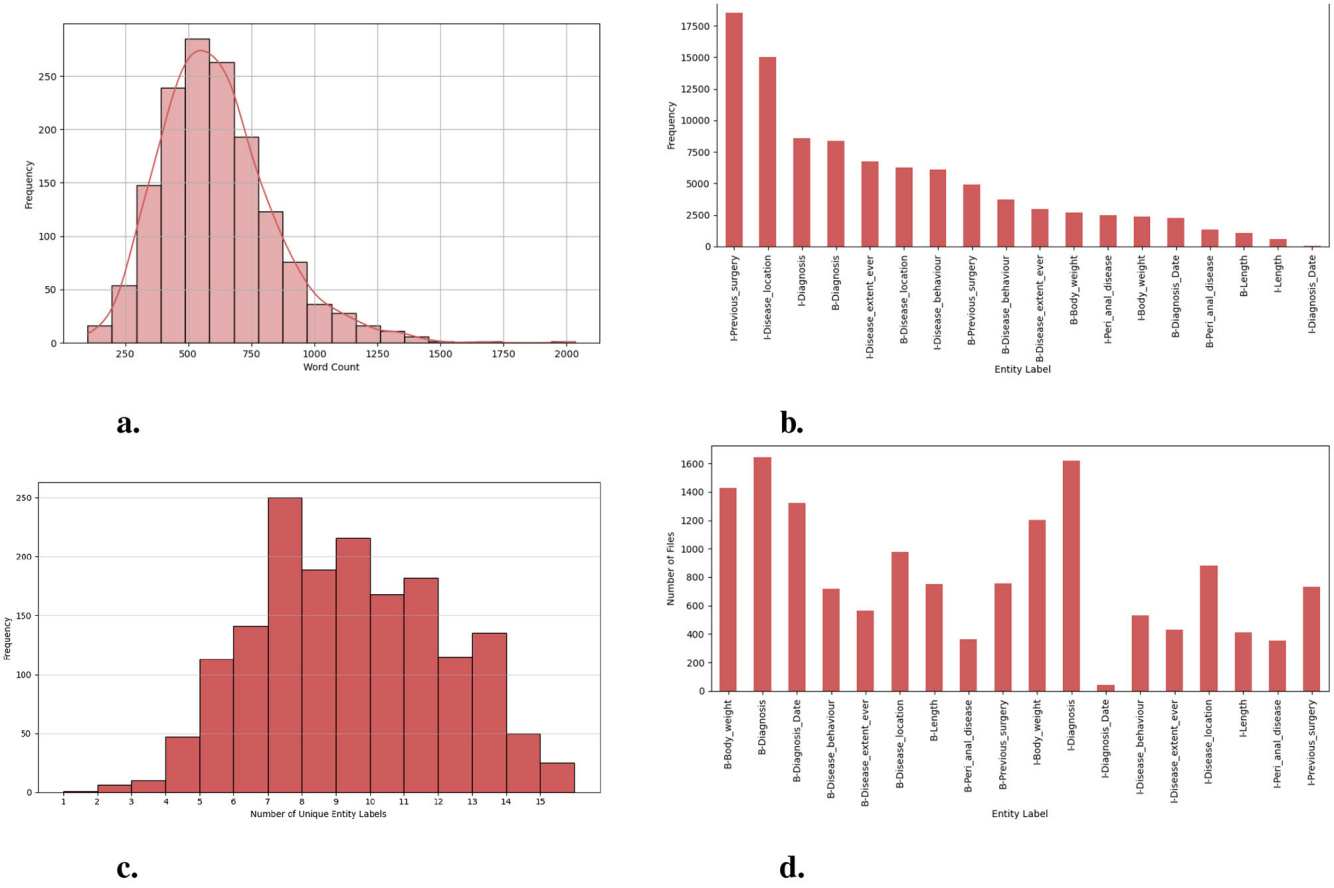
Exploration of dataset characteristics: Word and entity label distributions: **(A)** distribution of word counts in each clinical note; **(B)** frequency of unique entity labels [B-tags represent the start of an entity, while I-tags indicate the continuation of the same entity.]; **(C)** number of unique entity labels in each clinical note; and **(D)** frequency of clinical notes containing unique entity labels.

This research is motivated by the need for an efficient and comprehensive Clinical NER model that can automatically extract useful entity types of the IBD domain from free unstructured clinical texts. For this work, we are interested in identifying the important clinical entities related to Crohn's disease (CD) and ulcerative colitis (UC) cases like diagnosis, diagnosis date, body weight, length [height], previous surgery, disease location, disease behavior, disease extent, and peri anal disease. Refer to Figure 7 for entity-specific data distributions. As can be seen from Figure 7, the dataset is quite imbalanced with respect to the entity classes with entities like peri-anal disease, disease behavior, disease location, and previous surgery being the least commonly occurring entity in the clinical notes. These are also complex entities to detect because it span longer sequence lengths with multiple tokens and are often co-occurring in similar contexts. Table 1 shows the clinical entities of interest together with their descriptions in the IBD context.

**Table 1:** List of clinical entities relevant in the IBD domain together with their descriptions.

**Clinical entity**	**Description**
Diagnosis date	The date of diagnosis of disease
Diagnosis	The diagnosis which is either Crohn's disease (CD) or ulcerative colitis (UC) or variations of either CD or UC.
Disease location	According to Montreal classification can be either L1, L2, or L3: • L1: inflammation limited to the ileum • L2: inflammation limited to the colon segments (caecum-ascendus- descendus-sigmoid-rectum) • L3: inflammation of both ileum AND colon
Disease behavior	According to Montreal classification can be either B1, B2, or B3: • B1: non-stricturing, non-penetrating • B2: stricturing (leads to stenoses) • B3: penetrating (leads to fistula's and abscess)
Peri-anal disease	History of peri-anal disease
Previous surgery	Identify the type of surgery performed
Disease Extent	According to Montreal classification can be either E1, E2, or E3: • E1: inflammation limited to the rectum • E2: inflammation distal to the splenic flexure • E3: inflammation proximal to the splenic flexure
Length	Height of the person during consultation
Body weight	Weight of the person during consultation

### 5.1 Experiment setup

We use PyTorch and the HuggingFace library for the model implementation. We run our experiments on GPU: 1 × NVIDIA TITAN RTX with 24.0 GB RAM to integrate the components of our package. We use grid search to get the optimal values for the hyperparameters and use early stopping to overcome possible overfitting.

**EMLM finetuning:** We use the Dutch model (Delobelle et al., 2020) with an LM head to initialize the model parameters. EMLM is fine-tuned for 20 epochs using Adam optimizer (Kingma and Ba, 2015) with batch size set to 32 and learning rate set to 1e − 5 (with warm-up ratio of 10%).

**EMLM-CL finetuning:** After initializing the student and teacher, we continually pre-train the student on the same IBD clinical corpus for 150*k* steps. The training samples are truncated with a maximum length of 256 and the batch size is set as 32. The temperature parameter τ in Equation 3 is set as 0.01. We optimize the model with Adam optimizer with weighted decay and an initial learning rate of 1*e*−4 (with a warm-up ratio of 10%).

**NER model:** We use the Dutch model (Delobelle et al., 2020) with classification head as the NER model for all our experiments. We adopt Adamw optimizer (Loshchilov and Hutter, 2019) with a learning rate set to 5e − 5 and batch size set to 16. The NER model is trained for 10 epochs and the best model is selected according to dev set performance. The trained model is evaluated on test sets and we report the averaged Micro-F1 scores over 3 runs.

**Hyperparameter tuning:** In the domain continual pre-training schemes, we set the probability of masked language modeling to 0.15. The total epoch of the domain continual pre-training is 30. The masking rate η in EMLM fine-tuning is 0.7. All of these hyperparameters are tuned on the dev set with grid search. We add an early stopping mechanism to select the best model during the model training. The training will stop if the F1 score does not improve in 10 rounds. The dropout rate for the Roberta model is 0.1. The ϵ parameter of the label smoothing technique is 0.1. The model was trained on a single NVIDIA TITAN RTX GPU with 24 GB memory for 30 epochs, taking approximately 12 h. The model required an average of 50 ms per sample for inference, with a batch size of 16. The maximum GPU memory usage during training was 21 GB out of 24 GB, while inference required 4 GB.

### 5.2 Methods compared

To elaborate on the effectiveness of the proposed approaches for pre-training and fine-tuning, we compare the following method settings:

1. **Finetune-only (FT):** The NER model is trained on only the original training set annotated with clinical entities related to IBD.
2. **Finetune-fuse-section (FT-FS):** The NER model is trained on the original training set together with the section information fused into the model. Here clinical section information is additionally injected as external knowledge to the model.
3. **Finetune-fuse_section-pseudo_labels (FT-FS-PL):** The NER model is trained on the original training set together with the section information fused into the model. We further augment training data with the silver standard data obtained using pseudo-labeling. We also include Rdrop-based consistency training in the model training.
4. **MLM-Finetune-fuse_section-pseudo_labels (MLM-FT-FS-PL):** We randomly mask entity tokens and directly utilize a pretrained LM for MLM. Further, this model is fine-tuned on the training set.
5. **EMLM-Finetune-fuse_section-pseudo_labels (EMLM-FT-FS-PL):** We mask clinical entity tokens and insert the labels into inputs and utilize a pretrained LM to predict masked clinical entities using this context. Further, this model is fine-tuned on the training set.
6. **EMLM-Contrastive-Learning-Finetune-fuse_section-pseudo_labels (EMLM-CL-FT-FS-PL)**: The representations of masked entity tokens with labels inserted into input are contrastively learned against a teacher representation to learn more discriminative entity representation. Further, this model is fine-tuned on the training set.

## 6 Results and discussion

### 6.1 Evaluation metric

We follow the same validation metrics as previous NER approaches to evaluate the performance of our fine-tuned model on the human-annotated dataset. The validation metrics are precision (P), recall (R), and F1-score (F1) at the token level. Precision: percentage of clinical entities found by the model that is correct. Recall: percentage of clinical entities present in the data found by the model. F1-score: harmonic mean of precision and recall. Strict: A recognized entity is correct only if it is an exact match of the corresponding entity in the data file. For example: if the gold label of drug entity is *warfarin* and model prediction is *of warfarin*. The Strict match evaluation metric gives this an incorrect evaluation even if the type of entity (drug) is correctly recognized. Entity_Type: A recognized entity is correct if the entity type is correctly recognized but the exact surface boundary is missed. This metric holds particular importance in our case, as it gauges the model's ability to recognize clinical entities even when segmentation is not identical. This variability stems from differences in annotation styles and documentation practices among doctors.

### 6.2 Does continued pre-training help improve clinical domain adaptation?

**Figure 8:**
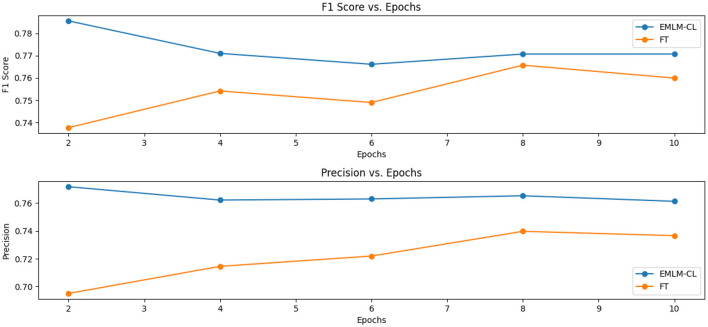
Exploring the influence of efficient pre-training on model fine-tuning: Demonstrating the advantage of pretraining in providing a strong initial representation for finetuning, even with minimal epochs.

Our results confirm with the previous research (Gururangan et al., 2020) that continued pretraining on domain-specific data indeed helps adapt the model to the target task. In comparison with the baseline model (FT) where we use a Dutch Roberta model for fine-tuning, we see that MLM-FT-FS-PL achieves better scores (72.84*%F*1 vs. 74.93*%F*1) which suggests that continued pre-training increases the overlap between the original LM and the target task domain. We further modified the MLM objective to include two additional pre-training strategies both of which drive the LM to focus on pivotal entities characterizing the domain at hand and to learn entity-centric knowledge. As can be seen from the results masking entity-specific knowledge together with inserting entity tags enhances the domain and task-specific knowledge of the LM. The goal of continued pre-training is to provide a better initial representation tailored to the domain at hand when fine-tuning a language model. As depicted in Figure 8, efficient pre-training strategies that learn better token-level representations provide a robust initial representation for finetuning, even with limited epochs. This is evident between the values in the F1 scores, with 73.75% F1 and 78.68% F1 scores observed between fine-tuned models and those fine-tuned from entity-centric in-domain pretrained models.

### 6.3 Which masking strategy provides a better-adapted LM?

**Figure 9:**
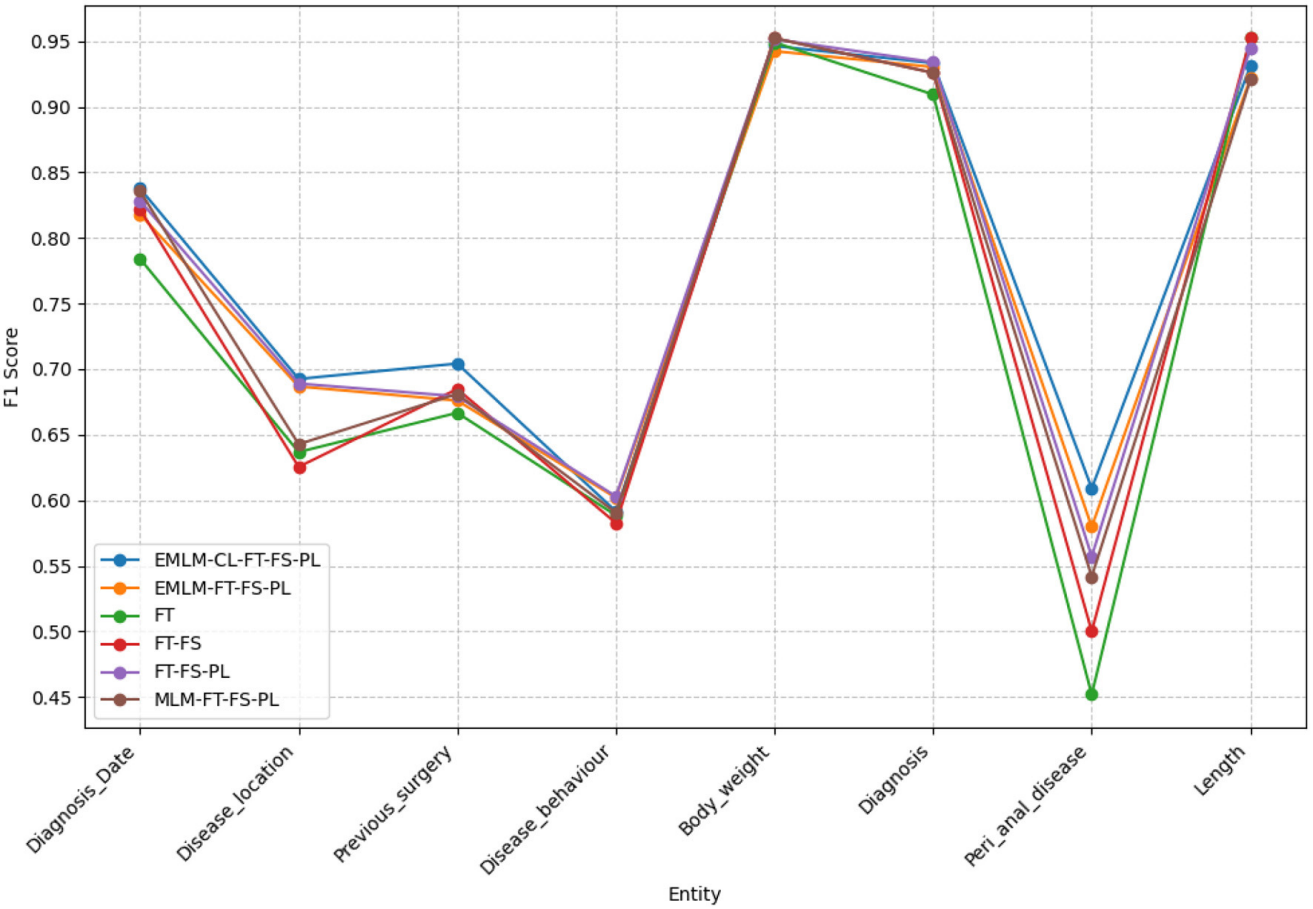
The F1-scores for various models examined across different clinical entities extracted within the IBD domain.

By comparing random masking (MLM-FT-FS-PL), entity-specific masking (EMLM-FT-FS-PL), and entity-specific masking with contrastive learning (EMLM-CL-FT-FS-PL), entity-masked contrastive objective helps the model learn better entity representations which make it the most suitably adapted LM for entity extraction task or in general for tasks requiring word-level information. Both entity masking strategies have significantly amplified the model's generalization capability, with an increased adaptation to the clinical IBD domain which is evident from the high precision (77.31%), recall (77.27%), and F1-score (77.29%) achieved by the model. Upon examining the F1 scores of individual entities depicted in Figure 9, it becomes evident that domain-specific entities with fewer annotations such as disease location, previous surgeries, and peri-anal disease which are diverse and challenging to detect, show notable enhancements in overall F1 scores with the implementation of entity-specific masking strategies also incorporating contrastive learning. For entities that have relatively more annotated examples and require less domain-specific context like body weight, length [height], and diagnosis date the difference is performance is minimal. We conducted an **ANOVA** (Girden, 1992) test to evaluate the statistical significance of the model results, yielding a *p*-value of 0.020183. Since *p* < 0.05, this confirms that there is a statistically significant difference in the means across the models. To further explore these differences, we performed a *post hoc* **Tukey HSD** (Honestly Significant Difference) (Keselman and Rogan, 1977) test, which allows for pairwise comparisons between all model groups. The analysis revealed that model FT differs significantly from models EMLM-CL-FT-FS-PL (*p*_adj_ = 0.0325) and EMLM-FT-FS-PL (*p*_adj_ = 0.0375).

### 6.4 Does section fusion information have an impact on fine-tuning performance?

The judgment of clinical entities cannot be done solely at the named entity level. For instance, “disease location” and “disease behavior” have different types of clinical significance in sections such as previous medical history and family medical history. The frequent use of domain-specific acronyms and abbreviations within different parts of a clinical note also increases the difficulty of entity extraction models. For example, in our case, Crohn's disease and Ulcerative colitis have abbreviations CD and UC/CU respectively. This acronym is also used in the laboratory section but indicates different measurements. Section headings can be seen as structured data once clinical documentation is complete which when injected into the NER model enhances contextual information to the NER model and helps reduce classification errors. This is evident in Table 2 that when inserting section information there is a significant improvement in the precision of the model compared to the baseline model (69.93% P vs. 74.09% P). Table 3 provides a few examples where the model predictions improve when injecting additional section title information during model fine-tuning.

**Table 2:** Methods and results: table reports the Precision (P), Recall (R), and F1 score (F1 score) of the different methods described.

	**Strict**			**Entity_Type**
**Method**	**P**	**R**	**F1**	**F1**
FT (Delobelle et al., 2020)	69.93	76.01	72.84 ±0.0011	83.79 ± 0.0013
FT-FS	74.09	76.22	74.62 ± 0.0020	85.01 ±0.0027
FT-FS-PL	76.25	76.93	76.71 ±0.00059	85.98 ± 0.0015
MLM-FT-FS-PL (Devlin et al., 2019; Lee et al., 2020)	72.51	**77.51**	74.93 ±0.0053	84.69 ±0.0027
EMLM-FT (Liao et al., 2024; Pergola et al., 2021)	75.32	76.51	76.01 ±0.0017	85.17 ±0.0013
EMLM-FT-FS-PL	76.75	77.17	76.86 ±0.0026	86.01 ±0.0010
EMLM-CL-FT-FS-PL	**77.31**	77.27	**77.29** **±0.0017**	**86.23** **±0.0007**

FT refers to the regular finetuning with the Dutch RoBERTa model for NER. FS refers to fusing section information into the NER model. PL denotes using pseudo labels and rdrop regularization in model training. EMLM refers to including entity-specific masking in the training and EMLM-CL refers to using entity-specific masking with contrastive learning. Bold values represent the highest values in each column of the results.

**Table 3:** Impact of incorporating section information attributes into the model- this table illustrates example predictions that were inaccurately made by the baseline model (FT), yet correctly disregarded by the model enhanced with section information fusion (FT-FS).

**Section title**	**Text**	**Predictions (FT)**	**Predictions (FT-FS)**
Family History (Familiaal)	Zus en nicht van moeder :, ziekte van Crohn., [translation: Mother's sister and cousin:, Crohn's disease.]	ziekte van Crohn (Diagnosis)	-
Family History (Familiaal)	Broers met Morbus crohn. [translation: Brothers with Crohn's disease.]	Morbus crohn (Diagnosis)	-

### 6.5 Does pseudo labels together with Rdrop help in improved generalization and performance?

Incorporating Rdrop and augmentations (pseudo-labeled examples) into NER improves the performance of the NER model as can be seen in Table 2. Pseudo-labeled data diversify and augment training data, boosting model robustness and generalization. Noise from augmented data reduces overfitting risk and the model's reliance on specific patterns. Augmented examples aid learning and capture linguistic nuances for better language understanding. Adding an Rdrop further enhances performance, akin to an ensemble of sub-models within a single model. These sub-models encourage the model to capture various aspects of the data and promote learning more generalized features. The model performance is improved by incorporating pseudo-labeled examples and Rdrop together with the structured section information (72.84% F1 vs. 76.71% F1). The variance in doctors' documentation methods highlights the disparity between the Strict and Entity_type F1-scores, underscoring the challenges in annotating entities. Greater consistency in annotation practices could help narrow this divergence.

### 6.6 Strengths and limitations

**Strengths:** The proposed method shows robust performance across a wide range of entity types, leveraging masked token prediction and contrastive learning to enhance feature representation. By masking entities and learning representations contrastively, the model effectively learns entity-context relationships, leading to improved accuracy in categories with rich contextual dependencies. Additionally, data augmentation helps balancing the representation of low-resource entities, and external information fusion further enriches the context for underperforming entity types. The approach is computationally efficient and scales well for datasets with diverse entity types, which is valuable in real-world applications with heterogeneous data.

**Limitations:** Accuracy differences between entity types suggest that the method still struggles with low-resource or less distinctive entities even though it largely improves from the baseline models. For example, entities with ambiguous or overlapping contexts may not be well-represented in the learned embeddings due to the lack of adequate examples. The efficacy of entity masking depends heavily on the choice of masking strategy, which might inadvertently bias the model toward overfitting certain entity types if not carefully designed. Leveraging external knowledge graphs or semantic embeddings can further optimize the context for underperforming entity types. Another step would be to implement masking strategies that adapt to the characteristics of specific entity types, providing more tailored learning signals.

### 6.7 Practical impact of the model in clinical applications

The model offers significant potential to enhance clinical workflows by analyzing unstructured clinical text, such as doctor's notes and lab reports, to extract and categorize key information relevant to Inflammatory Bowel Disease (IBD). This capability enables semi-automation of clinical documentation, while enabling the efficient extraction and organization of critical data. By identifying patterns within clinical data, the model can group patients based on disease severity, treatment response, or age. This grouping allows for more personalized and timely care.

While the current focus on Inflammatory Bowel Disease (IBD) clinical notes addresses a critical area, the underlying methods have the potential to be extended across other domains and languages. The methods could be adapted to analyze clinical notes for other chronic conditions, such as diabetes, cardiovascular disease, or rheumatoid arthritis. These conditions also generate large volumes of unstructured text, where automated entity extraction and categorization could streamline documentation and decision-making. Oncology clinical workflows could benefit from applying these techniques to extract tumor characteristics, treatment responses, and prognostic markers from unstructured text, aiding precision medicine. The methods can be tailored to analyze psychiatric evaluations, therapy notes, or patient-reported outcomes to detect patterns indicative of mental health disorders, providing support for early diagnosis and intervention.

Extending the model to multilingual settings can further transform IBD care by bridging language barriers and enabling its use across diverse regions. Multilingual adaptation enhances accessibility to advanced clinical NLP tools in IBD management. While this endeavor presents challenges, such as limited availability of data in under-represented languages, the initial steps can involve augmenting training datasets through machine translation or synthetic data generation. Collaborations with international healthcare institutions can provide access to diverse datasets, facilitating the development of robust multilingual clinical NLP systems. These efforts would ensure that the model remains adaptable and effective across linguistic and cultural contexts.

## 7 Error analysis

To understand why our model fails in some cases, we randomly select 50 error cases and group them into the 5 most prevalent error categories (see Table 4). Below, we elucidate the main error types with accompanying examples and compare predictions from different models (see Table 5).

1. **Segmentation error: missing information in predictions:** These instances occur when the model's predictions lack certain information. It is notable that often the overlooked text portions are not the most significant. This observation has been corroborated by domain experts. This often arises from variability in text complexity, where less significant portions of text are overlooked. We assume that refining the training data or using attention mechanisms to emphasize these segments could help mitigate such errors.
2. **Segmentation error: extra information in predictions:** The models sometimes extract additional information beyond the annotated text. This can stem from variability in patterns for entities with longer spans in text. Model experts regard this as a favorable feature of the model. While this reflects the model's ability to generalize, it is still a misalignment between annotations and predictions. Regularizing span detection and aligning annotation practices could improve this.
3. **Segmentation error: extraction of conjunctions:** Same entities in close proximity joined using some conjunctions are often extracted together. For instance, “*terminaal ileum en colitis tot 40 cm”* is extracted as a single disease location entity in model predictions, while annotations indicate “*terminaal ileum”* and “*colitis tot 40 cm”* as separate entities under disease location. This issue arises due to insufficient differentiation during training. Incorporating conjunction-based splitting rules could enhance entity boundary detection.
4. **Missing ground truth annotation:** Annotation text may be absent, yet the model correctly predicts it. This discrepancy can be attributed to annotation errors. Improving the annotation quality through iterative validation and human-in-the-loop review processes can address this discrepancy.
5. **Different entity predictions:** The model may predict a different entity type. This often occurs when there is a deviation from the typical annotation pattern. For example, “*Crohn colitis”* is annotated as a disease location entity whereas our model predicts “*Crohn”* as diagnosis and “*colitis”* as disease location. It should be noted that colitis is often regarded as a disease location during annotations. Enhancing training with more examples of these edge cases could reduce such errors.

**Table 4:** Error types: description of the main error types with examples showing the model predictions and true label.

**Model predictions**	**True Label**
▸***Segmentation error: Missing information in predictions***	
Disease location: rectum over 10 cm	Disease location: aantasting van het rectum over 10 cm [translation: involvement of the rectum over 10 cm]
▸***Segmentation error: Extra information in predictions***	
Disease location: rectitis ulcerohemorrhagica voor 5 cm [translation: rectitis ulcerohemorrhagica for 5 cm]	Disease location: rectitis
Disease location: erosies en ulcera verspreid over gans colon [translation: erosions and ulcers distributed throughout the colon]	Disease location: erosies en ulcera [translation: erosions and ulcers]
▸***Segmentation error: Extraction of conjunctions***	
Disease location: terminaal ileum en colitis tot 40 cm [translation: terminal ileum and colitis up to 40 cm ]	Disease location: terminaal ileum, Disease location: colitis tot 40 cm [translation: colitis up to 40 cm ]
▸***Missing ground truth annotation***	
Previous surgery: colectomie met tijdelijk ileostoma [translation: colectomy with temporary ileostomy]	-
▸***Different entity prediction***	
Diagnosis: Crohn, Disease location: colitis	Diagnosis: Crohn colitis

**Table 5:** Examples of predictions from different models with associated error types.

**Ground truth**	**FT (A)**	**EMLM-FT-FS-PL (B)**	**EMLM-CL-FT-FS-PL (C)**	**Error type**
*Terminaal ileum, colitis tot 40 cm [Translation: Terminal ileum, colitis up to 40 cm]*	*Terminaal ileum*	*Terminaal ileum en colitis tot 40 cm*	*Terminaal ileum en colitis tot 40 cm*	Under-prediction (A), merged predictions (B, C)
*Crohn (Diagnosis), colitis (Disease location)*	*Crohn colitis (Disease location)*	*Crohn (Diagnosis), colitis (Disease location)*	*Crohn (Diagnosis), colitis (Disease location)*	Entity misclassification (A)
*fistel en fisteltraject (disease behavior)*	*rectum (peri-anal disease)*	*rectum met fistel en fisteltraject (disease behavior)*	*excavatie in rectum met fistel en fisteltraject (disease behavior) [Translation: excavation in rectum with fistula and fistula tract]*	Entity misclassification (A), over-prediction (B,C)
*None*	*None*	*None*	*niet stenotische (Disease behavior) [Translation: non stenotic]*	Annotation error (C)
*inflammatoire angulatie in colon descendens op 30 cm (disease location)*	*colon (disease location)*	*colon descendens op 30 cm (disease location)*	*inflammatoire angulatie in colon descendens op 30 cm (disease location) [Translation: inflammatory angulation in descending colon at 30 cm]*	under-prediction (A,B)

FT refers to the regular finetuning with the Dutch RoBERTa model for NER. EMLM refers to including entity-specific masking in the training and EMLM-CL refers to using entity-specific masking with contrastive learning.

From Tables 4, 5, we see the five types of errors that occur when investigating the errors together with examples showing the model predictions and true labels. The errors justify the difference in the scores between Strict F1 and Entity-type F1 because the majority of errors occur due to differences in segmentation. This could be further improved with the help of our NER tool and a human-in-the-loop approach.

## 8 Conclusion

In conclusion, this paper has made significant contributions to the field of clinical Dutch named entity recognition (NER) within the context of inflammatory bowel disease (IBD). Through the development of a comprehensive pipeline encompassing annotation, pre-training, fine-tuning, and the creation of an end-to-end clinical Dutch NER tool, we have demonstrated the effectiveness of adapting a general Dutch language model to the specific domain of IBD.

Our work on developing a comprehensive pipeline for clinical NER in the IBD domain holds significant implications for the clinical setting in any domain or language or even a multilingual setting. By addressing the challenge of efficiently extracting vital patient information embedded in unstructured clinical text, our research directly impacts the way healthcare professionals interact with electronic health records (EHRs) and, consequently, patient care. The automated extraction of clinical concepts from unstructured text not only enhances communication among healthcare professionals but also contributes to improved patient care and a more thorough evaluation of healthcare outcomes. Our approach empowers medical practitioners, clinicians, nurses, and doctors with a highly accurate and efficient tool for swiftly retrieving relevant information from medical records. This not only saves time and energy previously spent on manual review but also ensures that clinicians have access to essential clinical information to make informed medical decisions.

Moreover, by leveraging domain and entity-aware masking and pre-training strategies, we ensure that our model excels in extracting relevant information related to the IBD domain. This adaptation enhances the model's ability to prioritize key domain-specific entities, ultimately improving its performance in the clinical setting. Our approach, which also incorporates iterative masking with pseudo-labels, and additional information fusion, and Rdrop-based consistency training, has yielded notable improvements in model performance. Furthermore, the integration of a multitask setting to simultaneously learn sentence-level categories and NER tags has shown promise in addressing challenges associated with low-performing entity classes.

The development of our NER tool further streamlines the information retrieval process for medical practitioners by providing options for annotating new notes, correcting existing annotations, and offering visualizations of predictions alongside true annotations. This tool enhances document readability, subsequently increasing the effectiveness of clinical experts in identifying key segments within medical records efficiently. However, we recognize that there is still room for improvement, particularly in leveraging structured patient information and multi-modal information and further refining the model's performance.

Our experiments have underscored the importance of entity-aware pre-training strategies, information fusion, and the utilization of pseudo-labeled data for silver standard data generation in enhancing entity extraction model performance. We envision that a human-in-the-loop approach, coupled with assistance from medical experts, particularly for entity classes with limited annotated data, could further elevate the model's performance toward human-level accuracy. Overall, our research not only contributes to advancing medical research through improved data extraction from EHRs but also directly impacts clinical workflows by providing clinicians with a valuable tool for enhancing patient care and medical decision-making. Our work shows that we can build a valuable information extraction tool for retrieving information from clinical reports even when only limited data that are manually annotated are available. Consequently, our work can be replicated in other clinical domains only requiring the limited effort of defining valuable entity types and of annotating a small set of clinical reports.

With an overall F1-score of 77.29% (strict) and 86.23% (ent_type) achieved on the clinical entity extraction task, despite the limited annotated data available for model training, our findings highlight the potential for continued advancements in this domain. Future work could explore a hierarchy of named entity labels as additional constraints that guide model training. It could even explore whether we can build a model for the recognition of general entity labels with the supervision of annotated data and learn their fine-grain categories in a fully unsupervised way without examples that were manually annotated as was done in Tian et al. (2024). Furthermore, continual learning (Wang et al., 2024) from human feedback and from the human corrections of entity extractions without retraining the full model is a promising avenue of future research where the model that continually learns should not forget the knowledge it has acquired previously. Additionally, we could leverage complementary information from multiple data modalities, such as text, images, and structured metadata, to enhance entity recognition in low-resource settings. Integrating visual and textual cues can disambiguate entities where textual data alone is insufficient, boosting accuracy. Techniques like cross-modal attention (Ye et al., 2019) mechanisms and shared embeddings enable efficient information fusion, allow models to learn robust representations from limited data. This approach could significantly improve performance in scenarios with scarce labeled resources or ambiguous contexts (Li et al., 2024). Finally, such a set-up could be combined with privacy-preserving federated learning (Truong et al., 2021) over different hospitals and services. Moving forward, we aim to refine our approach further, integrate structured patient information more effectively, and to continue collaborate with domain experts to push the boundaries of clinical NER research.

## Data Availability

The datasets presented in this article are not readily available because this dataset is a private dataset of the UZ Leuven hospital which is not yet approved to be used outside of the university site. Requests to access the datasets should be directed to Sumam Francis, sumam92@gmail.com.
